# Smoking, BCG and Employment and the Risk of Tuberculosis Infection in HIV-Infected Persons in South Africa

**DOI:** 10.1371/journal.pone.0047072

**Published:** 2012-10-09

**Authors:** Tolu Oni, Hannah P. Gideon, Nonzwakazi Bangani, Relebohile Tsekela, Ronnett Seldon, Kathryn Wood, Katalin A. Wilkinson, Rene T. Goliath, Tom H. M. Ottenhoff, Robert J. Wilkinson

**Affiliations:** 1 Clinical Infectious Disease Research Initiative, Institute of Infectious Diseases and Molecular Medicine, Faculty of Health Sciences, University of Cape Town, Cape Town, South Africa; 2 Division of Medicine, Imperial College London, London, United Kingdom; 3 Medical Research Council, National Institute for Medical Research, London, United Kingdom; 4 Leiden University Medical Centre, Leiden, The Netherlands; Hopital Raymond Poincare - Universite Versailles St. Quentin, France

## Abstract

**Background:**

The increased susceptibility to latent tuberculosis infection (LTBI) of HIV-1-infected persons represents a challenge in TB epidemic control. However few studies have evaluated LTBI predictors in a generalized HIV/TB epidemic setting.

**Methods:**

The study recruited 335 HIV-infected participants from Khayelitsha, Cape Town between February 2008 and November 2010. Tuberculin skin tests and interferon-gamma release assays were performed on all participants and active TB excluded using a symptom screen, TB microscopy and culture.

**Results:**

LTBI prevalence was 52.7% and 61.2% (TST and IGRA respectively). Being a recent TB contact (OR 2.07; 95% C.I. 1.15–3.69) was associated with TST positivity. Participants with a CD4>200 had a two-fold higher risk of IGRA positivity compared to those with CD4 counts <200 (OR 2.07; 95% C.I. 0.99–4.34). There was also a 19% increase in IGRA positivity risk for every additional year of schooling and a strong association between years of schooling and employment (p = 0.0004). A decreased risk of IGRA positivity was observed in persons with a BCG scar (OR 0.46; 95% C.I. 0.31–0.69) and in smokers (OR 0.47; 95% C.I. 0.23–0.96).

**Conclusion:**

We report the novel findings of a decreased risk of IGRA positivity in HIV-infected smokers possibly due to decreased interferon production, and in the persons with a BCG scar suggesting a protective role for BCG in this population. We also found an increased risk of TST positivity in employed persons, possibly due to ongoing transmission in public modes of transport.

## Introduction

A high prevalence of latent tuberculosis infection (LTBI) globally means there remains a pool of *M.tb.* infection from which new cases will arise. HIV-1 (HIV)-infected persons are often over-represented among TB cases, as the risk of re-infection and reactivation are greater.

Detection of LTBI enables identification of HIV-infected persons who would benefit from preventive therapy. However, in high HIV/TB prevalence, low-income settings, screening the general population for LTBI poses significant operational and economic challenges. A greater understanding of risk factors for infection could enable more accurate identification of high-risk persons who might benefit from preventive therapy. A better comprehension of factors that increase the risk of TB infection could also facilitate developing other interventions to attenuate this risk.

To this end, studies have examined the prevalence of, and risk factors for LTBI in health care workers (HCW), who experience a higher risk of TB exposure than the general population [1]–[11]. However, HCW do not represent the population of HIV-infected adults. The aim of this study was to describe the prevalence of LTBI and to evaluate associated risk factors in HIV-infected adults in a high HIV/TB burden setting.

## Methods

### Participants

From February 2008 to November 2010, consecutive patients attending the HIV wellness clinic at Khayelitsha day hospital were informed about the study on weekday mornings, when the clinic was busiest, and were invited to participate. Adults aged over 18 years with no signs and symptoms of TB disease were potentially eligible for inclusion. Active TB was excluded by induced sputum sent for TB microscopy and culture. In addition, all patients with a positive tuberculin skin test had chest radiography performed. During the study period 335 HIV-infected persons with no signs and symptoms of TB and from whom sputum samples could be collected, were identified. Written informed consent was obtained from all participants, and the study was approved by the University of Cape Town Faculty of Health Sciences Research Ethics Committee.

### Tuberculin Skin Test

A Mantoux skin test was performed in enrolled patients using 2 tuberculin units (TU) of purified protein derivative (PPD) of tuberculin RT23 injected intradermally into the volar aspect of the forearm. This was read 48–72 hours after administration by measuring the induration transverse diameter. A positive TST was defined as ≥5 mm.

### Interferon Gamma Release Assay (IGRA)

A 7-day in-house IGRA assay was performed as described elsewhere [12]. Briefly, whole blood was diluted 1∶10 final concentration in RPMI 1640 medium containing 1% L-Glutamine and added to a 24-well flat bottom plate at 1 ml/well. Antigens (ESAT-6, CFP-10 (Proteix, Czech Republic) and ESAT-6/CFP-10 Fusion Protein (Leiden University Medical Centre, Netherlands)) were added at a final concentration of 5 µg/ml. After mixing, the plate was incubated at 37°C with 5% CO_2_ for 7 days. The supernatants were then harvested into sample storage tubes and IFN-γ ELISA performed [13]. The antigen-specific cut-off values were chosen using the QuantiFERON Gold-in-Tube (QFT-GIT) test as an arbitrary gold standard. The lowest cut-off values that provided 100% sensitivity at the highest likelihood ratio were chosen.

### Statistical Analysis

Baseline characteristics were compared using simple proportions. Risk factors associated with TST and IGRA positivity were analysed using multivariable logistic regression. The model was built manually starting with an empty model. Nested models were compared using the likelihood ratio test. The Akaike’s Information Criterion (AIC) was used to compare non-nested models with a significantly lower AIC (>10%) indicating an improved model. Significance testing was done using a combination of two-sided p-values (p<0.05), 95% confidence intervals and p-value function graphs. All data were analysed using STATA 10.0 (StataCorp, College Station, TX, USA).

## Results

We recruited 335 HIV-infected persons. Compared to TST negative, TST positive participants were more likely to be employed (36.7% versus 29.3%), and were more likely to report contact with a TB case within the preceding 12 months (25.3% versus 14.1%) (Table 1). There was no difference in the median CD4 count between TST negative and positive persons (CD4 337 (IQR 226–337) versus 362 (IQR 253–519); Spearman’s correlation coefficient r = 0.082; p = 0.148)). However, the median overall CD4 count of 345 in this study population reflects a lack of severe immunosuppression and could explain the lack of an association between CD4 count and TST positivity. Alternatively, the study may also have been insufficiently powered to detect this association.

**Table 1:** Baseline characteristics stratified by TST status. TST tuberculin skin; N sample size; IGRA interferon gamma release assay; TB tuberculosis.

Characteristics		TST Negative N (%)	TST Positive N (%)	Total N (%)
Sex	Female	130 (86.7)	139 (83.2)	269 (84.9)
	Male	20 (13.3)	28 (16.7)	48 (15.1)
IGRA	Negative	62 (42.2)	23 (14.0)	85 (27.2)
	Positive	68 (46.3)	122 (73.9)	190 (60.9)
	Indeterminate	17 (11.5)	20 (12.1)	37 (11.9)
Smoker	No	131 (87.3)	144 (86.8)	275 (87.0)
	Yes	19 (12.7)	22 (13.2)	41 (13.0)
Recent TB contact	No	128 (85.9)	121 (74.7)	249 (80.1)
	Yes	21 (14.1)	41 (25.3)	62 (19.9)
Previous TB	No	123 (82.6)	146 (87.4)	269 (85.1)
	Yes	26 (17.4)	21 (12.6)	47 (14.9)
BCG Scar	No	61 (41.2)	82 (49.1)	143 (45.4)
	Yes	87 (58.8)	85 (50.9)	172 (54.6)
Employed	No	106 (70.7)	105 (63.3)	211 (66.8)
	Yes	44 (29.3)	61 (36.7)	105 (33.2)
Accommodation	Shack	99 (66.4)	90 (57.0)	189 (61.6)
	House	50 (33.6)	68 (43.0)	118 (38.4)
Time resident in Khayelitsha	<1 year	7 (4.7)	12 (7.6)	19 (6.2)
	> = 1 year	141 (95.3)	145 (92.4)	286 (93.8)
On Antiretroviral therapy	No	148 (99.3)	163 (98.2)	311 (98.7)
	Yes	1 (0.7)	3 (1.8)	4 (1.3)
Characteristics		Median (IQR)	Median (IQR)	Median (IQR)
Age (years)		30.6	31.6	31
		(27.5–36.9)	(26.8–37.5)	(26.8–37.5)
Body Mass Index (kg/m^2^)		27.9	26.6	26.8
		(23.3–31.6)	(22.6–31.3)	(22.9–31.4)
Education	Highest school grade achieved	11 (10–11)	11 (9–12)	11 (9–12)
Number of persons/bedroom		2 (1.67–3)	2 (1.67–3)	2 (1.67–3)
CD4 count (cells/mm^3^)		337	362	345
		(226–476.5)	(253–519)	(241–504)
Number of days since HIV diagnosis		410.5	337	351
		(12–1474)	(25–1316)	(19–1402)

### Prevalence of TST and IGRA Positivity

We examined the LTBI prevalence using the TST and found a prevalence of 52.7% (95% C.I. 47.3%–58.3%). Using the IGRA, the effect of immunosuppression was less marked with a higher prevalence of IGRA positivity compared to TST positivity (61.2% (95% C.I. 55.7%–66.4%).

### Factors Associated with TB Infection

A recent contact with a TB case in the preceding 12 months was the only significant variable associated with TST positivity in the multivariable regression model (OR 2.07; 95% C.I. 1.15–3.69). The inclusion of additional variables into this model did not significantly improve the fit of the model to the data.

**Figure 1:**
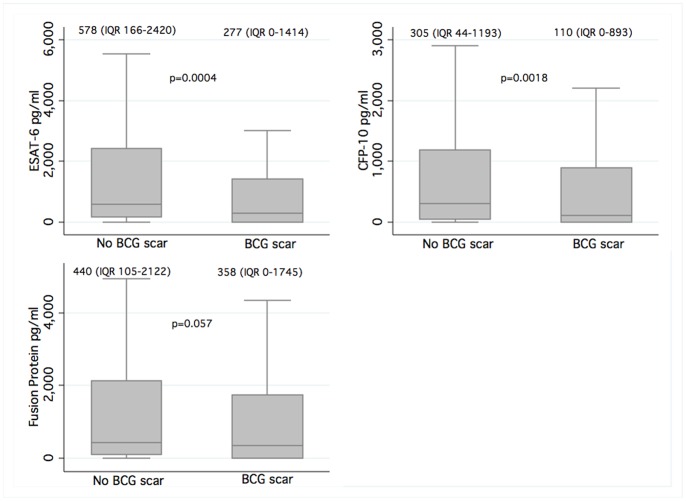
Box and whiskers plots showing quantitative IGRA antigen responses (pg/ml) stratified by presence or absence of BCG scar in the overall study population. Medians and interquartile ranges shown above each plot. Extreme values not shown.

The presence of a BCG scar was associated with a reduction in risk of IGRA positivity (OR 0.59; 95% C.I. 0.35–1.02) (Table 2). The overall median ESAT-6 (277 (IQR 0–1414) vs. 578 (IQR 166–2420)), CFP-10 (110 (IQR 0–893) vs. 305 (IQR 44–1193)) and fusion protein (358 (IQR 0–1745) vs. 440 (IQR 105–2122)) responses (pg/ml) were lower in persons with a BCG scar compared to those without a scar respectively (Figure 1). In addition, there was an increase in the risk of IGRA positivity for every additional year of schooling (OR 1.19; 95% C.I. 1.05–1.34). Of note, there was a strong correlation between educational attainment and employment status (Pearson chi^2^ p = 0.005).

**Table 2:** Final model of risk factors associated with IGRA positivity.

IGRA	Odds Ratio	95% C.I.	P-value
CD4> = 200	2.07	0.99–4.34	0.054
On ART	0.10	0.01–1.02	0.052
Highest school grade achieved	1.19	1.05–1.34	0.005
BCG scar	0.59	0.35–1.02	0.057
Smoker	0.47	0.23–0.96	0.038

The odds of being IGRA positive were 53% lower in smokers compared to non-smokers. We considered misclassification bias as a potential reason for this finding and performed sensitivity analysis, creating a new smoking variable by re-classifying ex-smokers as current smokers. This resulted in an increase in the proportion of female smokers from 24% to 28%. However, this did not alter the final model results. We also examined the interaction between smoking and gender, and found a statistically significant interaction variable suggesting that the effect of smoking on LTBI risk is different for the two gender groups, with a higher risk of IGRA positivity in male smokers (interaction variable OR 3.12; p = 0.038). We also compared the mitogen responses between smokers and non-smokers and although lower in smokers, there was no statistically significant difference in the median response to mitogen in smokers (11338 (IQR 5611–27606) pg/ml) versus non-smokers (12832 (IQR 3873–34179) pg/ml).

The odds of being IGRA positive were 2-fold higher in HIV-infected persons with CD4 count ≥200 cells/mm^3^ compared to <200 cells/mm^3^. Although only 2% of HIV-infected persons were on ART, we found that the odds of being IGRA positive in persons on ART were 90% lower than those not on ART, after adjusting for CD4 count.

Although the CD4 count, ART status and BCG scar variables in this model had p values of >0.05, the corresponding p-value function graphs showed that majority of data lay away from the line of no effect. The CD4 count and BCG scar graphs were also narrow, indicating high precision of estimates (graphs not shown).

## Discussion

We examined the prevalence of latent TB infection and risk factors that associate with TST and IGRA in HIV-infected adults in a high HIV/TB-burden setting and interestingly these factors differed.

TST positivity was associated with a recent TB contact history. Few TB contact studies have been conducted in high HIV/TB burden settings [14], [15] and these support data from this study showing a higher risk of TB infection among TB contacts with the risk increasing with duration and intensity of exposure [15].

Our results revealed decreased odds of IGRA positivity in smokers compared to non-smokers. Existing literature has shown an increased risk of TST positivity in smokers and so this finding was unexpected [16]. Smoking is known to have immunosuppressive effects and has also been shown in *in vitro* studies to significantly reduce monocyte derived macrophage production of interferon-gamma [17]. These effects combined with HIV infection, although increasing the risk of TB infection, could paradoxically result in decreased odds of IGRA positivity, potentially limiting the performance of IGRA in HIV-infected smokers.

We also found increased odds of IGRA positivity with more years of education. Education was strongly associated with employment in our data suggesting these variables may be measuring similar exposures. The risk of LTBI is conventionally thought to be higher in lower socioeconomic groups and several studies conducted in high burden settings have reported increased risk of TB with lower income [18], [19]. We therefore expected unemployment (as a marker of marginalisation and lower socioeconomic status) to be associated with a higher risk of LTBI. However, the data suggest the opposite. Khayelitsha is an informal township 30km outside Cape Town and employment necessitates travel to Cape Town using overcrowded and poorly ventilated modes of public transport. Furthermore the force of infection in Cape Town townships has previously been reported as being exceptionally high [20]. One possible explanation is that TB transmission is occurring on public transport increasing the risk of LTBI in employed persons who use these modes of transport more frequently and for longer distances and are potentially repeatedly exposed. Alternatively, these results could indicate exposure to TB from co-workers or in the general work environment, although the majority of employment opportunities occur in more affluent parts of Cape Town, where the TB burden is significantly lower.

There was a negative association between IGRA positivity and the presence of BCG scar. These findings suggest a protective role for BCG against LTBI diagnosed using IGRA. In high-burden settings, BCG vaccination is given at birth to reduce the risk of disseminated TB in children. These results suggest that BCG also protects against TB infection in HIV co-infected young adults. This effect of BCG against LTBI has been reported in one study of children with household TB contacts that reported the absence of a BCG scar as an independent risk factor for LTBI diagnosed using an in-house ELISpot assay [21].

### Limitations

BCG vaccination status was determined using the presence of a BCG scar. However it is possible that some participants were BCG vaccinated but had no scars. Therefore the protective effect of BCG might be limited to persons who scar. Another possibility proposed by Lalvani *et al* is that the scarring response to BCG could represent a surrogate marker of individuals who have pre-existing protective immunity to tuberculosis that is not induced by BCG vaccination [22].

The use of an in-house IGRA limits the generalisability of these findings. Quantitative antigen responses were therefore presented and demonstrate significantly lower responses in persons with a BCG scar. Furthermore, an earlier comparison of this assay with the QFT-GIT revealed an identical proportion of positive responders suggesting comparability between these tests.

There is a possibility of bias due to misclassification of smokers. We performed sensitivity analysis and showed that the final result was unchanged. We however acknowledge residual confounding as a potential limitation of this study.

### Conclusion

Our data indicate an increased risk of LTBI in TB contacts highlighting ongoing transmission in the community. The novel finding of a higher risk of LTBI in educated and employed persons also suggests ongoing transmission and further research is required to explore the contribution of public transport to high transmission rates and the potential for public space to be targeted in the development of TB control strategies in high-burden settings. Smoking, as a risk factor for LTBI, is important from a public health perspective, with up to 15% of TB cases worldwide estimated to be attributable to tobacco exposure [23]. It is a potentially more readily modifiable risk factor compared to other factors that predispose to TB infection and disease and therefore a potential target for TB control strategies. We found a lower rate of IGRA positivity in HIV-infected smokers. This may relate to the immunosuppressive effects of smoking and impaired interferon-gamma production in HIV-infected persons, potentially limiting the performance of this test in this sub-group. A recent study of 1128 children in Europe showed that BCG vaccination may be effective at protecting children against *M.tb.* infection [24]. We found the presence of a BCG scar to be negatively associated with IGRA positivity suggesting a protective role for BCG vaccination in HIV co-infected adults.
